# Natural killer (NK) cells with downregulated activating receptors and IL-10 production promote *Trypanosoma cruzi* T-cell responses in subjects in the Chronic phase of *Trypanosoma cruzi* infection: An exploratory immunological analysis

**DOI:** 10.1371/journal.pntd.0014622

**Published:** 2026-08-12

**Authors:** María J. Elias, Gonzalo Cesar, María G. Alvarez, Ariel Podhorzer, María B. Caputo, Constanza Lopez Albizu, Máximo Carrega, Flavio A. Tomán Conte, María A. Natale, María C. Albareda, Bruno Lococo, Susana A. Laucella

**Affiliations:** 1 Instituto Nacional de Parasitología “Dr. Mario Fatala Chaben”, Buenos Aires, Argentina; 2 Chagas disease Unit. Hospital Interzonal General de Agudos Eva Perón, Buenos Aires, Argentina; 3 GENYO. Center for Genomics and Oncological Research. Pfizer/University of Granada/Andalusian Regional Government, Granada, Spain; KARI-Trypanosomiasis Res Centre, KENYA

## Abstract

**Background:**

Subjects with chronic Chagas disease and no signs of heart disease exhibit decreased NKp46 expression, high CD57 expression, and IL-10 production by NK cells. This study provides a detailed characterization of the phenotype and function of NK cells according to the severity of heart disease, and evaluates how these changes following treatment with benznidazole, as well as their association with *Trypanosoma cruzi*-specific T-cell responses.

**Methods:**

The phenotype and function of NK cells in a cohort of 51 subjects infected with *Trypanosoma cruzi* and exhibiting varying degrees of heart disease were evaluated using high-dimensional flow cytometry and Boolean gating analysis. ELISPOT assays were performed to measure IFN-γ and IL-2 production in response to *T. cruzi* antigens, using CD56, CD4 and CD8-depleted PBMC.

**Results:**

In contrast to individuals without heart disease, those with advanced cardiomyopathy have an increased number of NK cells that express the activating receptors NKp46 and CD16, as well as the differentiation marker CD57. NK cells in subjects with advanced cardiomyopathy were also found to be enriched in CD56^+^CD107^+^granzyme B^+^TNF-α^+^ cells and depleted of CD56^+^IL-10^+^ cells. The function of NK cells shifted towards monofunctionality in subjects with declining *T. cruzi*-specific antibodies following treatment with benznidazole. Eliminating CD56^+^ cells significantly decreased the number of IFN-γ-producing cells in response to *T. cruzi*.

**Conclusions:**

In chronic *T. cruzi* infection, NK-cell function may be balanced by the downregulation of activating receptors and IL-10 production, However, parasite persistence may desensitize the regulation of NK activating receptors, enabling NK cells to exert a potent polyfunctional cytotoxicity that potentially induce tissue damage.

## Author summary

While T cells are known to play a key role in the immune response against *Trypanosoma cruzi*, the contribution of natural killer (NK) cells—immune cells that provide early defense against pathogens and help to regulate immune responses—remains unclear. In this study, we evaluated the NK-cell phenotype and function in patients with different clinical forms of chronic Chagas disease and examined how they are affected by etiological treatment, as well as their association with *Trypanosoma cruzi*-specific T-cell responses. We found that individuals without cardiac disease exhibited NK cells with reduced expression of activating receptors and increased production of the regulatory cytokine IL-10, suggesting a role in controlling excessive immune responses. By contrast, patients with cardiomyopathy exhibited NK cells with an exacerbated functional profile. We also found evidence that NK cells support parasite-specific T-cell responses and that successful benznidazole treatment is associated with a shift toward a resting status of these cells. Together, these results highlight NK cells as important modulators of immunity and disease progression in chronic Chagas disease.

## Background

Chagas disease is a potentially life-threatening illness that affects approximately 6–7 million people [1,2]. Initially, the disease was confined to endemic areas, primarily in Latin America, but due to increased population mobility in recent decades, most infected individuals now reside in urban areas. Consequently, the infection has been increasingly detected in non-endemic countries, transforming it into a global public health concern [2–4].

*T. cruzi*-specific T cell responses in chronic Chagas disease are critical for infection control [5]. A low level of detectable *T. cruzi*-specific T cells, along with a predominance of monofunctional cytokine-producing T cells, is found in the circulation of subjects with long-term *T. cruzi* infections, particularly those with more advanced cardiac disease [6]. Innate immune cells play an integral role in the host's defense against pathogens. Among them natural killer (NK) cells served to control the burden of intracellular pathogens by lysing infected cells or producing effector cytokines until adaptive immune responses develop in acute infections [7]. NK cells may also exert regulatory functions on T cells [8,9], and thus may play a key role also in chronic infections [10,11].

The function of NK cells is regulated by the integration of activating and inhibitory receptors. Reduced or weakened signals from activating receptors allow inhibitory signals to dominate, thereby decreasing NK-cell functionality [12,13]. Several reports have demonstrated that, in the context of chronic infections, NK cells exhibit a state of exhaustion similar to that of exhausted T cells, displaying altered function and phenotype [12–14].

We have shown that NK cells in subjects with chronic Chagas disease who have not yet developed cardiac alterations exhibit decreased expression of the activating receptor NKP46, high expression of the differentiation marker CD57 and produce IL-10 [15] supporting a regulatory role of NK cells in chronic Chagas disease.

The present study evaluated the relationship between adaptive and innate immune responses in relation to cardiac dysfunction, and how these responses are modulated by etiological treatment. The hypothesis to be assessed is whether NK-cell function becomes exhausted in more severe stages of chronic *T. cruzi* infection, or whether it is exacerbated.

## Methods

### Ethic statement

This study was approved by the Institutional Review Board of the Hospital Interzonal General de Agudos Eva Perón, Buenos Aires, Argentina (Memorandum No. 19/19). All patients signed informed consent forms prior to inclusion in the study.

### Selection of study population

Patients who tested positive for at least two out of three serological tests for *T. cruzi* infection (i.e., indirect immunofluorescence assays, hemagglutination and ELISA tests), known as “conventional serology”, were considered to be infected [2]. Subjects were clinically evaluated and grouped according to a modified version of the Kuschnir grading system [16,17]: group 0 (G0), seropositive individuals exhibiting a normal electrocardiogram (ECG) and normal echocardiograph; group 1 (G1), seropositive individuals with a normal echocardiograph but abnormalities in the ECG; group 2 (G2), seropositive individuals with ECG abnormalities and heart enlargement; and group 3 (G3), seropositive individuals with ECG abnormalities, heart enlargement and clinical or radiological evidence of heart failure. Groups 2 and 3 were presented together because both groups showed evidence of cardiac muscle compromise, as confirmed by echocardiography. When treatment with benznidazole was indicated, this was administered at 5mg/kg body weight per day for30days [18]. All the subjects that were treated belonged to the G0 group. Monitoring of *T. cruzi*-specific antibodies were performed at 6 months post-treatment and once a year thereafter up to 48 months of follow-up. Treatment was considered successful when at least one of the following criteria for conventional serology was fulfilled — conversion of positive serology to negative results on three or two tests performed and decrease in titers on at least 2 tests out of the three tests (i.e., 30% reduction in ELISA titers, and a 2-fold dilution by indirect immunofluorescence assays or hemagglutination) [17,19] — or when a 50% reduction in the reactivity of at least two proteins in the Luminex-based, multiantigen serological assay was observed [17,20,21]. Unchanged *T. cruzi*-specific antibody levels post-treatment, positive findings using qPCR [22,23], or disease progression during post-treatment follow-up was considered treatment failure. Age-matched uninfected subjects from Buenos Aires who have always resided in nonendemic areas and with negative serological findings for *T. cruzi* infection served as the uninfected control group. The study population characteristics are summarized in Table 1.

**Table 1:** Baseline characteristics of study population in *Trypanosoma cruzi*-infected subjects and uninfected controls.

Patient group	n	Mean age +	Gender		Serology for *T. cruzi*	Cardiological findings (n)	
		SD (years)	Male	Female		Electrocardiography	Echocardiography
G0	16	40 + 6	8	8	Positive	N (16)	N (16)
G1	13	45 + 12	7	6	Positive	LAFB (2); CRBBB (9); CAVB (1); PM (2)	Apical aneurysm (1); inferobasal hypokinesia (1); mild systolic function decrease (1)
G2/G3	9	56 + 6	7	2	Positive	CRBB (4); LAFB (4); PM (3); AFIB (2)	Inferobasal hypokinesia (1); mild to severe LVDD increase (8); left ventricular apical aneurysm with thrombus (1); moderate to severe systolic function decrease (6); global hypokinesia (1)
Benznidazole-treated	13	38 + 7	8	5	Positive	N (13)	N (13)
Uninfected	15	42 + 12	6	9	Negative	N (15)	N (15)

AFIB (arial fibrillation); CAVB (complete atrioventricular block); CRBBB (complete right bundle branch block); LAFV (left anterior fascicular block); LVDD (left ventricular diastolyc diameter); PM (pace maker); N (normal) Groups 2 and 3 were presented together because both groups showed evidence of cardiac muscle compromise, as confirmed by echocardiography

### Collection of peripheral blood mononuclear cells (PBMC)

Approximately 50 mL of blood was drawn by venipuncture into heparinized tubes (Vacutainer, BD Biosciences) and PBMC isolated as previously described [24].

### NK-cell phenotype and high dimensional analysis

One a half million PBMC were stained with a combination of monoclonal antibodies specific for CD56, CD16, CD3, CD57, CD127, KIR2DL2, NKp46, NKG2D and a dump channel for CD14 and CD19 with the addition of a viability dye [S1 Table]. The gating strategy for the selection of CD56^bright^ and CD56^dim^ NK-cell subsets is shown in S1.

Approximately 500,000 events were acquired per sample in a FACSAria II flow cytometer (BD Biosciences, USA). A high-dimensional analysis was carried out that consisted of the following steps: FlowAI (v.2.3.1) was used to discard anomalous events (setting: all checks, second fraction FR = 0.1, alpha FR = 0.01, maximum changepoints = 3, changepoint penalty = 200, and dynamic range check side = both). Then, the CD56^+^ NK population was selected using a manual gating strategy, excluding doublets, dead cells and CD3^+^ lymphocytes. DownSample (v.3.3) was used to even the number of events represented by each FCS file. Clinical parameter categorical values for each sample were added to downsampled populations as metadata to enable identification of these groups, and these were then concatenated for analysis. A dimension reduction analysis was performed using tSNE. For dimension reduction and clustering algorithms, all parameters were analyzed except for live/ dead NIR, CD3 and CD56. Those samples that had mean fluorescence intensity (MFI) values higher than two standard deviations away from the parameter mean (calculated from all samples) in two or more of the parameters considered for the high-dimensional analysis were excluded from the study to ensure the consistency of the analysis. An arbitrary cut-off point was set, whereby samples in which the number of events in the CD56^bright^ and CD56^dim^ NK-cell subpopulations did not reach 300 and 2,500, respectively, were excluded from the analysis. For analysis of the data, only clusters over 2.5% frequency were selected.

### NK-cell function

For NK-cell function, 3x10^6^ PBMCs were cultured with phorbol 12-myristate 13-acetate (PMA, 10 ng/ml, Sigma‒Aldrich) and ionomycin (1 μg/ml, Sigma‒Aldrich) in the presence of brefeldin A (10 μg/ml, Sigma‒Aldrich) for 4 hours [15,25]. Anti-CD107a was added at the onset of incubation. Unstimulated PBMC did not receive any treatment and served as controls. Surface staining was conducted using anti-human antibodies specific for CD56, CD16 and CD3, with the addition of a viability dye [S1 Table]. Following permeabilization using Cytofix/Cytoperm buffer (BD Biosciences), staining was conducted using a cocktail of antibodies specific for granzyme B, interferon (IFN)-γ, interleukin (IL)-10 and tumor necrosis factor (TNF)-α [S1 Table]. Unstained and fluorescence minus one (FMO) sample were used as gating controls. Approximately 600 000 events were collected per sample in the FACSAria II flow cytometer, and data were analyzed by FlowJo software (Version 10.8). Cytokine coexpression profiles of CD56^+^ NK cells were determined using the Boolean gating function of FlowJo software. Background responses detected in unstimulated control samples were subtracted from those detected in PMA/ionomycin-stimulated samples for every functional combination. NK cell responses were considered positive when cytokine production in the stimulated samples was at least double that obtained with the media alone, and when at least five events, or at least three events for single and polyfunctional responses, were present [15].

### ELISPOT assays following CD56 or CD4/CD8 depletion

Ten million PBMC were incubated with anti-human CD56 magnetic particles (BD Biosciences) according to the manufacturer’s instructions and the depleted fraction was collected using a cell separation magnet. This process was repeated twice, after which the cell suspensions were counted [S2 Fig]. A second fraction of 1 x 10⁷ PBMC was depleted using a mixture of anti-human CD4 and anti-human CD8 particles (BD Biosciences) [S3 Fig]. Whole PBMC, as well as those depleted of CD56 and CD4/CD8, were stained with monoclonal antibodies to CD56, CD4, CD8 and CD3 to measure the efficiency of depletion. Afterwards, ELISPOT assays were conducted using a commercial kit (ELISPOT Human IFN-γ or IL-2 ELISPOT Set; BD Biosciences, USA), as described elsewhere [26]. Briefly, whole or depleted PBMC, were seeded at a concentration of 4x10^5^ cells/well and stimulated with 10 µg/mL *T. cruzi* lysate from the Brazil strain or RPMI for 16–20 hr at 37°C and 5% CO2. Spot forming cells (SFCs) were automatically enumerated using ImmunoSpot analyzer (CTL). The mean number of spots in duplicate wells was obtained for each condition, and the number of specific IFN-γ and IL-2-secreting T-cells was calculated by subtracting the value of the wells containing media alone from the antigen-stimulated spot count. Responses were considered positive if (1) a minimum of 10 SFC/4 × 10^5^ PBMC were present per well and (2) this number was at least twice the value of wells with media alone [26].

### Statistics

Normal distribution was assessed using the Shapiro–Wilk test. The Kruskal-Wallis test and analysis of variance were used for multiple comparisons. Changes in cytokine producing NK cells post-treatment were evaluated using principal component analysis (PCA). The correlations between variables was determined by a Spearman or Pearson test, as appropriate. Two-tailed P < 0.05 were considered statistically significant. Statistical analysis was conducted using Analytical Software Statistix 10.0 and Graphpad 10.4.1

## Results

### NK-cell phenotype according to the severity of chronic Chagas disease

**Fig 1:**
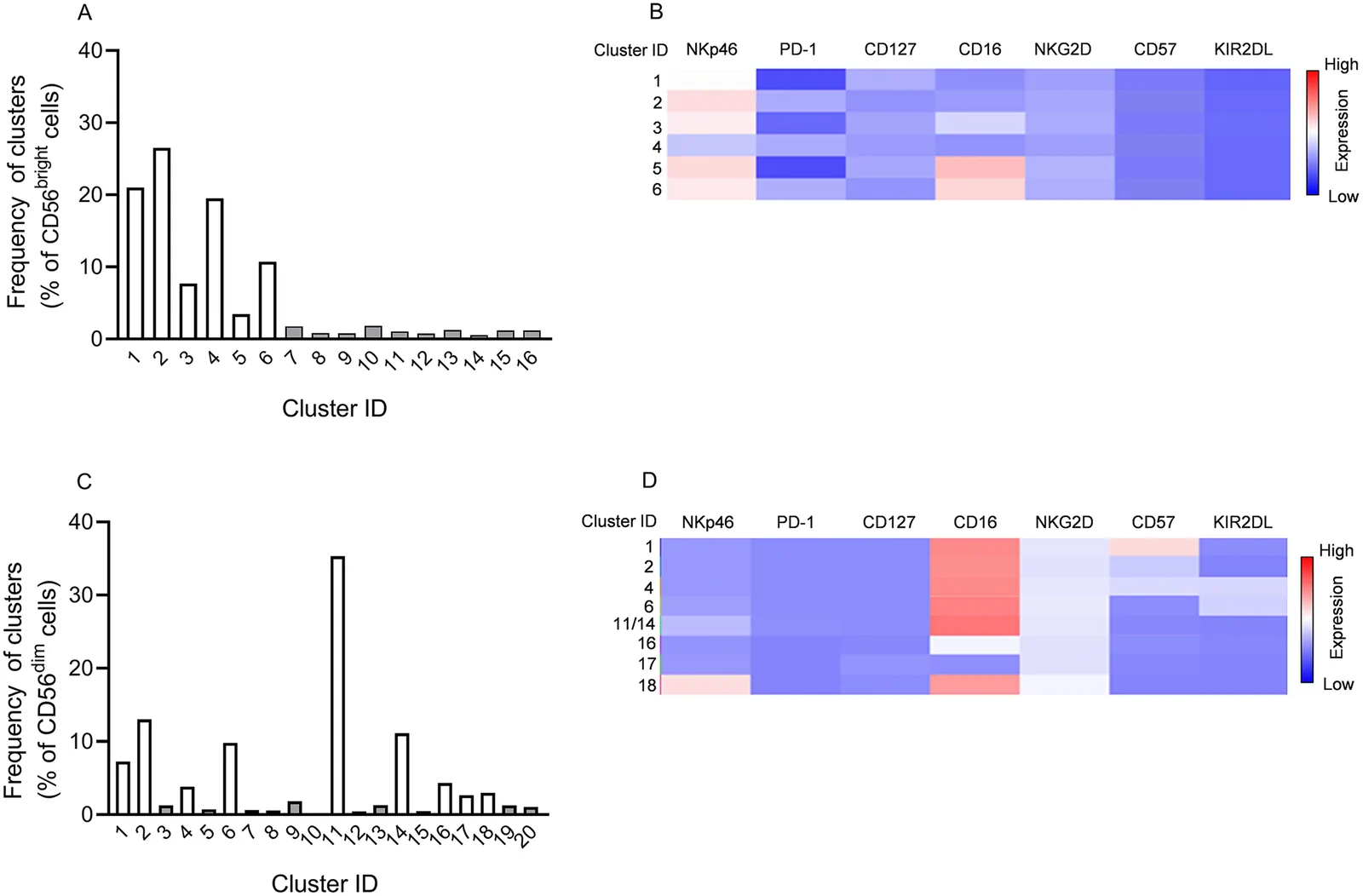
Unsupervised high-dimensional analysis of CD56^+^ NK-cell phenotype in subjects with chronic *T. cruzi* infection. PBMC were stained with a panel of monoclonal antibodies specific for surface markers and adquired by flow cytometry. Phenograph followed by FlowSOM clustering algorithms was applied on PBMC samples of *T. cruzi*-infected subjects (G0, n = 11; G1, n = 7; G2-G3, n = 9), and uninfected subjects (n = 15) The percentages of the CD56^bright^ (A) and CD56^dim^ (C) clusters that are higher or lower than 2.5% are shown in white or grey, respectively. Hierarchical clustering heatmaps showing the expression of the indicated proteins in the clusters identified on pre-gated CD56^bright^ (B) or CD56^dim^ (D) NK cells.

**Fig 2:**
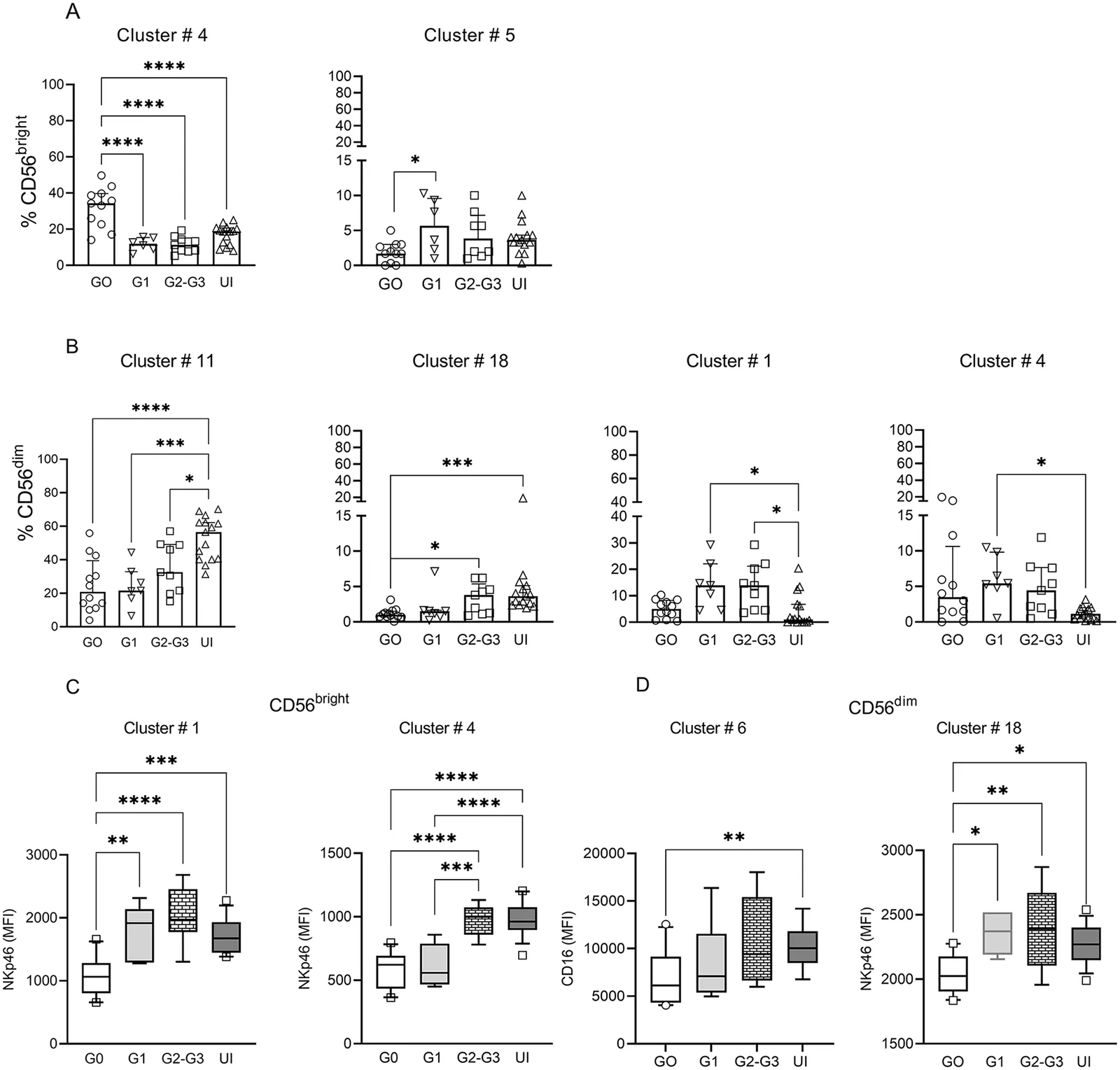
Unsupervised high-dimensional analysis of CD56^+^ NK-cell phenotype in *T. cruzi* infected subjects according to disease severity. Phenograph followed by FlowSOM clustering algorithms was applied on PBMC samples (G0, n = 11; G1, n = 7; G2-G3, n = 9; uninfected controls, UI, n = 15). Percentages of individual CD56^bright^ (A) or CD56^dim^ (B) clusters. Expression of the mean fluorescence intensity (MFI) for individual surface markers (C-D). Data are presented as median with interquartile range (A and B) or box and whiskers with 10-90 percentile (C-D). Differences among groups were evaluated by ANOVA or the Kruskal–Wallis test followed by posttests. **** P < 0.0001, *** P < 0.001, ** P < 0.01, * P < 0.05.

Regardless of their clinical status, *T. cruzi*-infected subjects had decreased levels of CD56^bright^ and increased percentages of CD56^dim^ compared with seronegative subjects [S4A and S4B Fig and S2 Table]. The NK-cell phenotype was evaluated based on the expression of the following markers: activating receptors (NKG2D and NKp46); inhibition (KIR2D); immaturity (CD127); differentiation (CD57); exhaustion (PD-1) and cytotoxicity (CD16) on CD56^bright^ and CD56^dim^ NK-cell subsets [15,27]. An unsupervised analysis was performed using the Phenograph tool, followed by FlowSOM clustering after concatenating all samples from all clinical groups. This identified 16 clusters of CD56^bright^ [Fig 1A] and 20 clusters of CD56^dim^ [Fig 1C] NK cells expressing one or more of the selected markers. Six of these clusters were found in CD56^bright^ [Fig 1B] and nine in CD56^dim^ [Fig 1D] NK cells, with a mean abundance greater than 2.5% and were selected for further analysis. S3 Table and S4 Table summarize the findings of the heat maps, which show the relative expression of each marker in each cluster, as well as their functional roles. Subsequently, the data were divided into clinical groups. It was found that, compared with patients with heart disease (groups G1 and G2/G3) and uninfected controls, subjects in the G0 group without cardiac alterations showed an increased percentage of CD56^bright^ NK cells with low expression of the activating receptor NKp46 and no expression of the cytotoxic receptor CD16 (cluster #4 in Fig 1B and S3 Table; Fig 2A and S5 Table), as well as a decreased percentage of CD56^bright^ NK cells with high expression of NKp46 and CD16 (cluster ≠5 in Fig 1B and S3 Table; Fig 2A, S5 Table). Conversely, subjects with mild (group G1) or advanced heart disease (group G2/G3) showed increased percentages of CD56^dim^ cells co-expressing the activating receptor NKG2D and the differentiation marker CD57 [cluster #1 in Fig 1D and S4 Table; Fig 2B and S5 Table], as well as the inhibitory receptor KIR2DL2 [cluster #4 in Fig 1D and S4 Table; Fig 2B, S5 Table], compared with uninfected subjects. Regardless the clinical stage, *T. cruzi*-infected individuals showed decreased percentages of CD56^dim^NKG2D^+^CD16^+^ [cluster # 11 of Fig 1D and S4 table, Fig 2B, S5 table] However, a significant linear trend in the percentage of cluster # 11 was observed alongside increasing disease severity (P < 0.0001). Subjects in the G0 group exhibited decreased levels of CD56^dim^NKp46^+^NKG2D^+^CD16^+^ [cluster # 18 of Fig 1D and S4 Table, Fig 2B, S5 Table] NK cells, than to those with severe heart disease or uninfected subjects.

**Fig 3:**
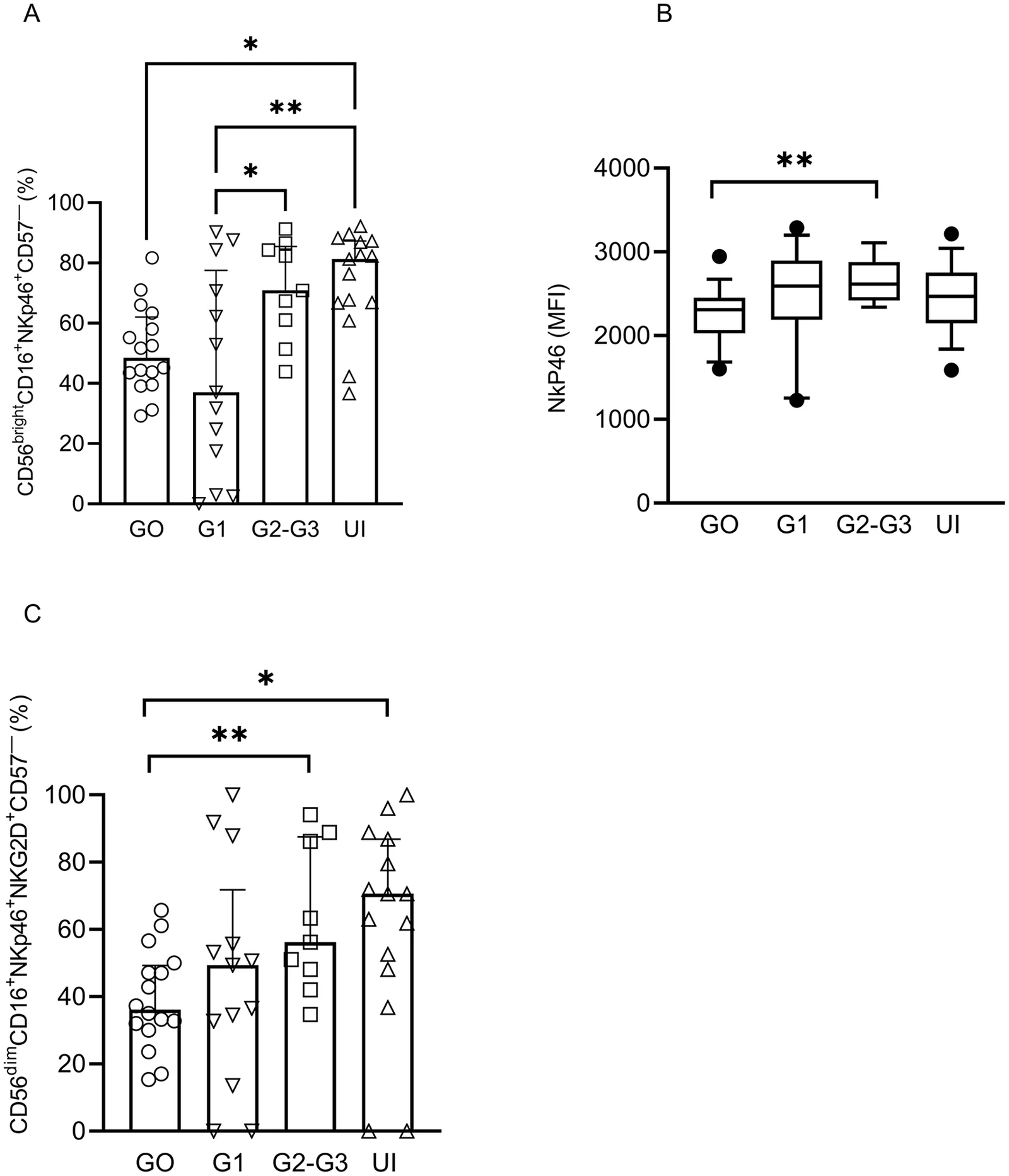
Manual gating strategy for the identification of NK cells with activating and cytotoxic phenotype. PBMC from *T. cruzi*-infected subjects (G0, n = 16; G1, n = 13; G2-G3, n = 9) and uninfected controls (UI, n = 15) were stained with a panel of monoclonal antibodies specific for NK cell surface markers and adquired by flow cytometry. Manual gating was used to select CD56^bright^CD16^—^ (A) and CD56^dim^CD16^+^ (C) NK cells, which express NKp46/NKG2D but not CD57. (B) Expression of the mean fluorescence intensity (MFI) of NkP46 in A. Data in A and C are presented as median with interquartile range and as box and whiskers in B. Differences among groups were evaluated by ANOVA or the Kruskal–Wallis test followed by posttests. ** P < 0.01, * P < 0.05.

Analysis of mean fluorescence intensity of the different markers in each cluster showed that patients with more advanced heart disease exhibited higher expression of NKp46 in CD56^bright^[Fig 2C, clusters #1, #4 of Fig 1B, S3 Table and S6 Table] and CD56^dim^ [Fig 2D, cluster #18 of Fig 1D and S4 Table] as well as CD16 in CD56^dim^ NK cells [Fig 2D, cluster # 6 of Fig 1D and S4 Table and S6 Table] compared with those with no signs of heart disease. The manual gating strategy confirmed that, compared to subjects with advanced heart disease [right panel of S5 Fig], those with no or mild heart disease had decreased percentages of CD56^bright^CD16^+^NKp46^+^CD57^—^[Fig 3A, left panel of S5 Fig and S7 Table] and CD56^dim^CD16^+^NKp46^+^NKG2D^+^CD57^—^ NK cells [Fig 3C, right panel of S5 Fig and S7 Table] as well as lower NKp46 expression [Fig 3B]

### NK-cell function according to the severity of chronic Chagas disease

**Fig 4:**
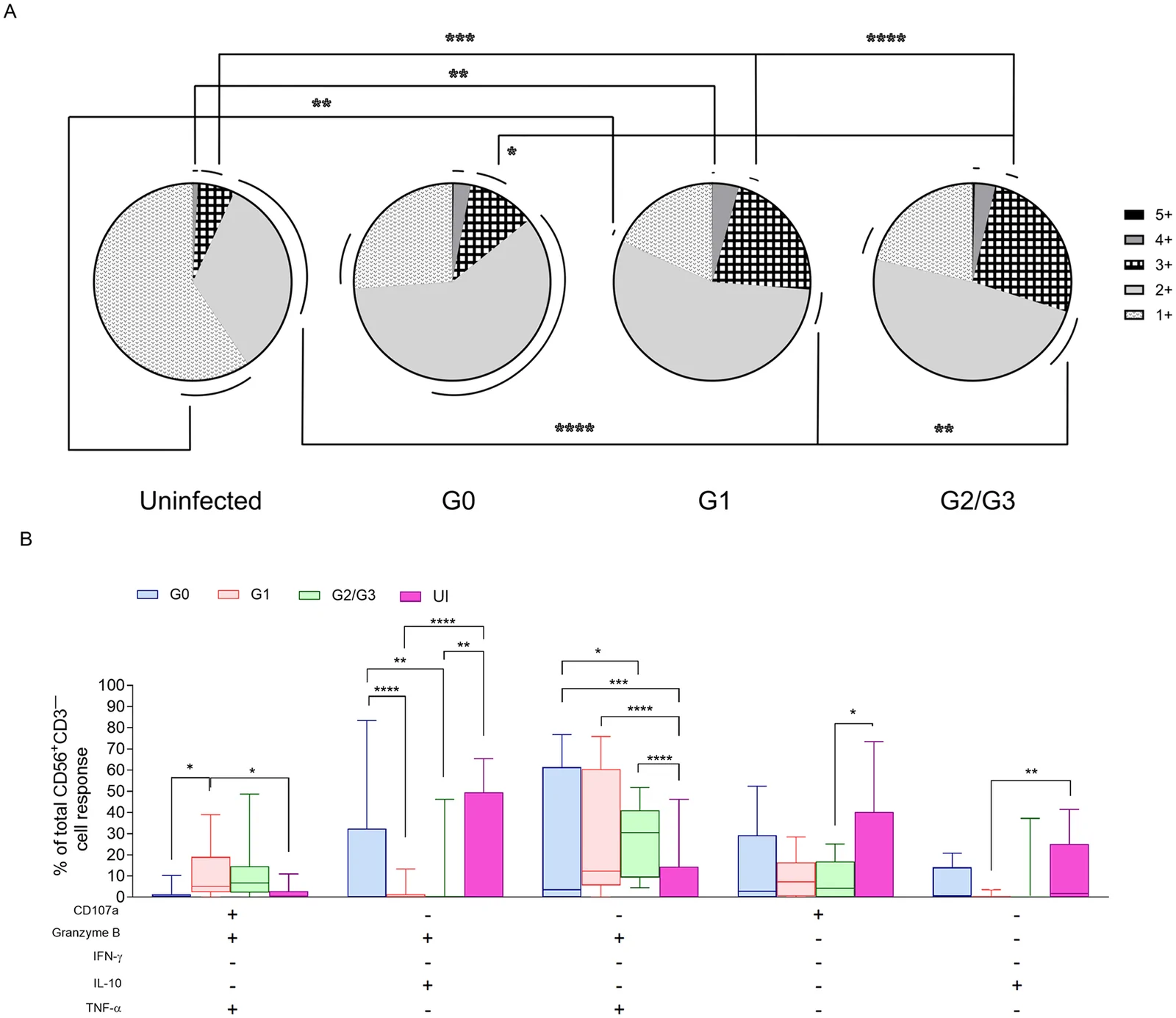
NK-cell function in *T. cruzi*-infected subjects with different degrees of heart disease. PBMC of *T. cruzi*-infected subjects in the G0 (n = 16), G1 (n = 13), G2/G3 (n = 9) clinical groups, as well as from uninfected subjects (UI, n = 15), were stimulated with PMA/ionomycin for 4 h and analyzed by flow cytometry for intracellular expression of CD107a, granzyme B, IFN-γ, IL-10 and TNF-α. A. Lymphocytes were gated based on forward scatter (FSC) and side scatter (SSC) parameters, and analysed for viable CD14^—^CD19^—^CD3^—^CD56^+^ cells versus each marker using Zombie Aqua. The coexpression profiles of one to five functions were identified using the Boolean gating function of the FlowJo software. The contribution of each molecule-producing subset to the total NK-cell response was assessed in donors with positive responses in the intracellular staining assay as indicated in the Materials and Methods. Then, the average for each combination out of 31 possible combinations was calculated. (A) The data are summarized by the pie charts, in which each slice of the pie represents the fraction of the total response that consists of CD56^+^ T-cells positive for one to five functions. The arches indicate the proportion of CD56^+^ T-cells that express IL-10 in each slice of the pie. (B) The bars indicate the percentage of the total response contributed by CD56^+^ NK cells with a given co-expression profile. The results are presented as box and whiskers with 10-90 percentile. Only the cytokine subsets showing significant differences between clinical groups out of the 31 analyzed are depicted. The values obtained in unstimulated samples were subtracted. Differences among groups were assessed using a Kruskal–Wallis test, with p values adjusted for multiple comparisons using Dunn's posttest. *. **** P < 0.0001, *** P < 0.001, ** P < 0.01, * P < 0.05.

We then evaluated the function of NK cells in the different clinical groups using Boolean gating analysis after stimulation with PMA/ionomycin which enabled us to categorize cytokine-producing NK cells into 31 different subsets, each consisting of one to five cytokine-producing populations. Subjects with more advanced heart disease (groups G1 and G2–G3) showed higher percentages of NK cells exhibiting four or three functions, compared to subjects without cardiac disease (group G0) [Fig 4A]. NK cells with two, three or four functions in subjects infected with *T. cruzi* and suffering from mild or advanced heart disease were found to be deprived of IL-10-producing cells, compared with subjects infected with *T. cruzi* but not suffering from heart disease, and uninfected subjects [see arches in Fig 4A]. Significant differences were found in five of the 31 possible co-expression profiles when the contribution of each subset to the total NK-cell response was analyzed individually. Patients with cardiomyopathy (groups G1 and G2-G3) had lower percentages of CD56^+^granzyme B^+^IL-10^+^, monofunctional CD56^+^IL-10^+^ and monofunctional CD56^+^CD107a^+^ NK cells than subjects with no signs of heart disease or infection [Fig 4B and S8 Table]. Conversely, NK cells in patients with cardiomyopathy were enriched in CD56^+^CD107^+^granzyme B^+^TNF-α^+^ and CD56^+^CD107^+^ TNF-α^+^ NK cells [Fig 4B and S8 Table]. Of note, the percentage of monofunctional CD56^+^IL-10^+^ cells was found to be inversely correlated with the levels of cluster #11 of CD56dim NK cells, which express NKp46 and NKG2D (R = −0.44, P = 0.04), while CD56^+^ cells with dual function (granzyme B^+^IL-10^+^) were found to be inversely correlated with CD56dim cells expressing NKG2D (R = −0.47, P = 0.03).

### NK-cell function after treatment with benznidazole

**Fig 5:**
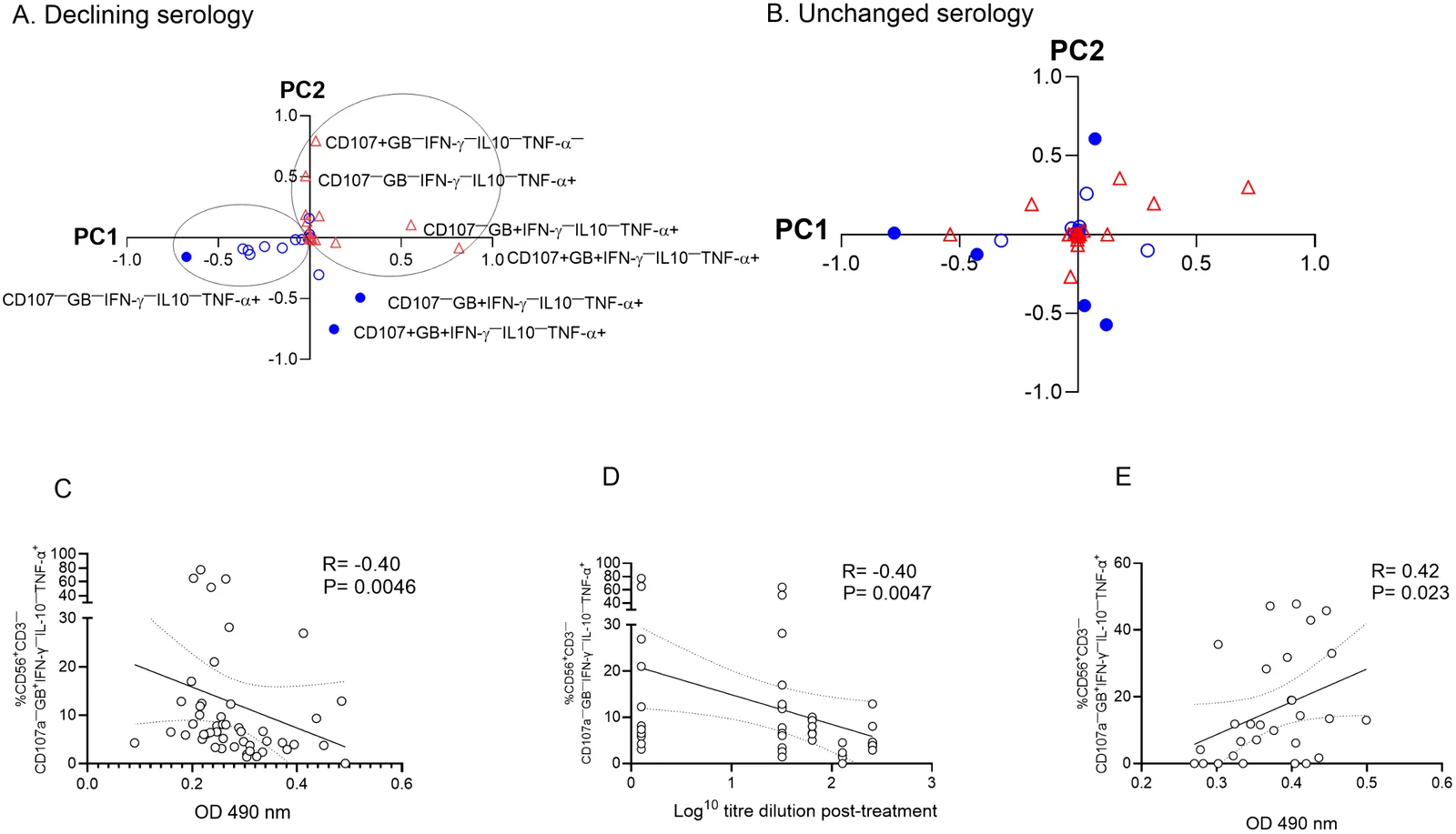
Principal component analysis of NK-cell function in *T. cruzi*-infected subjects following benznidazole treatment. PBMC were stimulated with PMA/ionomycin for 4 h and analyzed by flow cytometry for intracellular expression of CD107a, granzyme B, IFN-γ, IL-10 and TNF-α. Coexpression profiles with one to five functions using the Boolean gating function of FlowJo software. The contribution of each molecule-producing subset to the total NK-cell response was assessed in donors with positive responses in the intracellular staining assay as indicated in the Materials and Methods. Then, the average for each combination was calculated. The values obtained in unstimulated samples were subtracted. Following log transformation, the principal components were extracted with eigenvalues of 1.0. Factor loadings >0, 4 or <-0.4 were considered as influential mediators (filled symbols). NK cell functional profiles plotted based on the first 2 extracted principal components PC1 and PC2 prior to treatment (circle symbols) or following 48 months post-treatment with benznidazole (triangle symbols). *T. cruzi*-infected subjects with declining (A, n = 8) and or unchanged (B, n = 5) *T. cruzi*-specific antibodies following 48 months post-treatment. The Spearman test was applied to assess the correlation between the percentages of monofunctional and polyfunctional CD56^+^CD3-CD107-GB-IFN-gamma-IL-10-TNF-alpha^+^^—^ cells and *T. cruzi*-specific antibody levels measured by ELISA (C and E) and hemagglutination (D) in subjects with declining (C and D) or unchanged (E) serology for *T. cruzi* infection posttreatment.

PCA analysis was conducted to evaluate differences in NK-cell cytokine expression profiles in chronic Chagas disease patients before and after treatment with benznidazole. NK-cell cytokine expression cytokine profiles in PC1 could distinguish before and after treatment status in subjects with declining *T. cruzi*-specific antibodies after benznidazole treatment [Fig 5A]. The PC1, PC2 loading factors indicate that in this analysis differential expression of CD107^+^granzyme B^+^IFN-γ^—^IL-10^—^TNF- α^+^, CD107^+^granzyme B^—^IFN-γ^—^IL-10^—^TNF-α^—^, CD107^—^granzyme B^+^IFN-γ^—^IL-10^—^TNF-α^+^ and CD107^—^granzyme B^—^IFN-γ^—^IL-10^—^TNF-α^+^ strongly influences segregation of pre-treatment from post-treatment cytokine profile in these subjects (e.g., loading factor >0.4 or <−0.4). Conversely, PCA analysis of NK-cell cytokine expression profiles was unable to separate pre-treatment and post-treatment status in subjects with unchanged serological titers after treatment, albeit undetectable parasite DNA [Fig 5B]. The levels of *T. cruzi*-specific antibodies in subjects with declining serology post-treatment were inversely associated with monofunctional TNF-α-secreting NK cells [Fig 5C and 5D], whereas in subjects with unaltered serology post-treatment, they were positively associated with dual-function granzyme B^+^TNF-α^+^ NK cells [Fig 5E].

### Contribution of NK cells to the T-cell response specific for T. cruzi,

**Fig 6:**
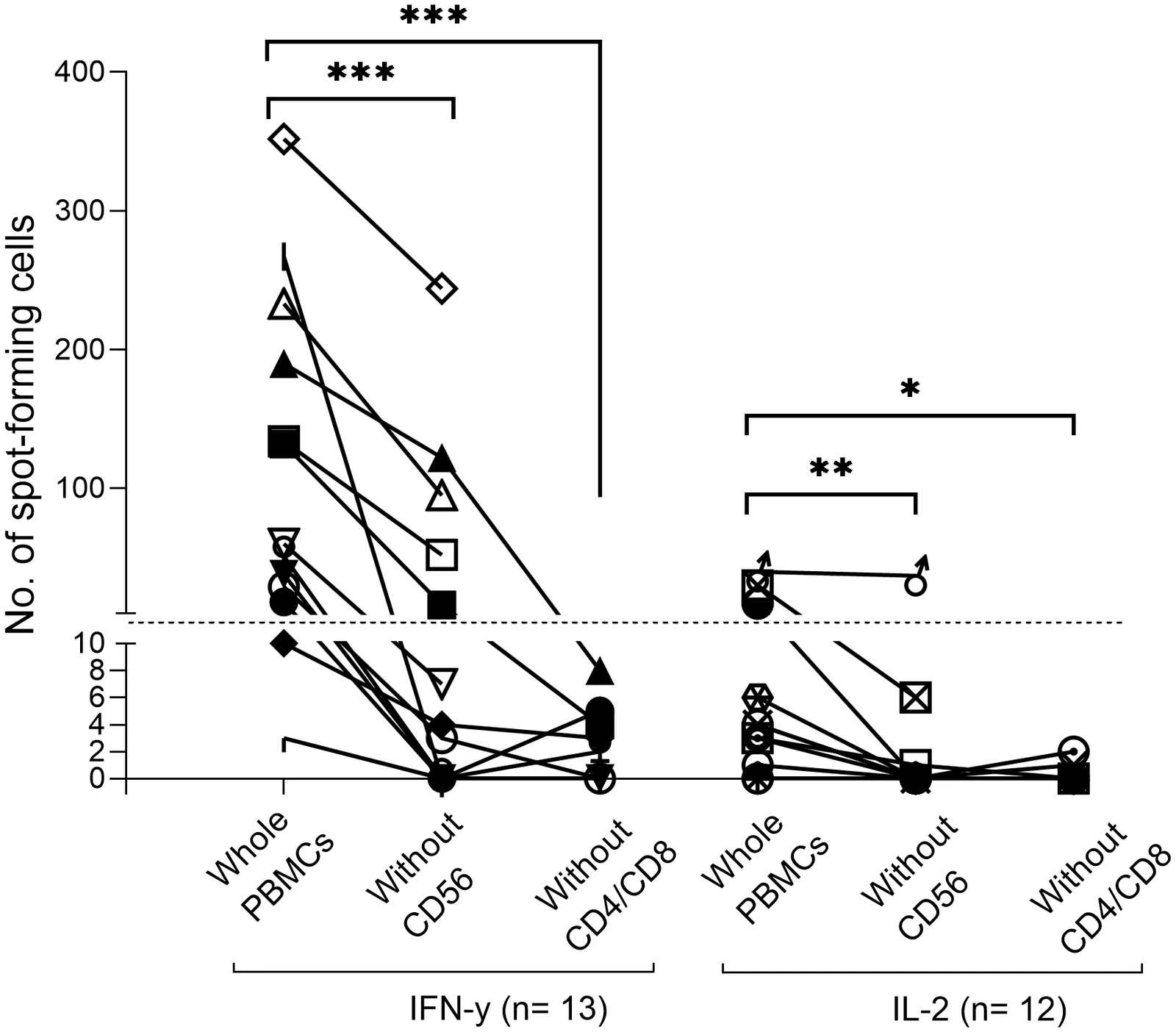
No. of interferon IFN-γ-secreting cells in response to *T. cruzi* lysate before and after depletion of CD56^+^ NK cells. Whole PBMC, CD56-depleted PBMC or double CD4 and CD8 depleted PBMC using magnetic beads were seeded at a concentration of 4x10^5^ cells/well and stimulated with 10 mg/mL *T. cruzi* lysate from the Brazil strain for 16–20 hours. Each point represents the mean number of spots in duplicate wells for each patient and experimental condition, connected by a line.* P < 0.05,**P < 0.01, ***, P < 0.001.

T-cell responses specific for *T. cruzi* in subjects with chronic Chagas disease are impaired in terms of both function and magnitude [24,28,29], but it remains unclear whether CD56^+^ (NK/NKT) cells may regulate these responses. To address this, the production of IFN-γ and IL-2 by PBMC from *T. cruzi*-infected subjects with no signs of cardiac disease was evaluated in response to a *T. cruzi* lysate, in the presence or absence of CD56^+^ cells. Due to the low frequencies of *T. cruzi*-specific T cells in the chronic phase, the ELISPOT technique was the most suitable method for addressing this question. The elimination of 85% of CD56^+^ cells, on average, [S2 Fig] resulted in a significant decrease in the number of *T. cruzi*-specific IFN-γ-producing cells [Fig 6]. To rule out the possibility that the decrease was due to IFN-γ production by CD56^+^ cells, an enriched sample of CD56^+^ cells, obtained after the depletion of CD4^+^ and CD8^+^ T lymphocytes (with average depletions of 90% and 82%, respectively; [Fig 3), was stimulated with *T. cruzi* lysate, and the number of IFN-γ- and IL-2-producing cells was measured. Enrichment of the CD56^+^ sample was found to be skewed towards NK cells, and the number of IFN-γ-producing cells was found to be below the positivity cut-off level for the ELISPOT technique [Fig 6]. Although the levels were lower than IFN-γ, the production of *T. cruzi*-specific IL-2 also decreased in the absence of CD56^+^ cells [Fig 6].

## Discussion

Here, we demonstrate distinctive phenotypic and functional signatures in NK cells from different chronically *T. cruzi*-infected patient groups. The downregulation of activating receptors in NK cells of subjects infected with *T. cruzi* without signs of heart disease contrasts with the profile observed in NK cells of subjects with heart disease, which are enriched in cells expressing activating, cytotoxic and differentiation NK receptors, alongside low IL-10 production and polyfunctional NK cells skewed towards cytotoxic capacity. In addition, a decline in *T. cruzi*-specific antibody levels following benznidazole treatment was associated with an increase in monofunctional NK cells.

The induction and potency of NK-cell responses depend on their activation state, which is regulated by signals transmitted through activating and inhibitory receptors. Early after infection, the inflammatory response may lead to the overexpression of activating receptors on NK cells or their ligands on target cells [30] positively modulating dendritic cell function and secreting cytokines that promote Th1 cell responses. The chronic engagement of NK receptor ligands by NK cells may result in the modulation of activating receptors, thereby limiting NK-cell cytotoxic potential. Several mechanisms have been described, such as downregulation of activating receptors [31–35], downregulation of activating receptor ligands [36] and shedding of the activating receptors themselves [37,38]. The down-modulation of activating NK-cell receptors may depend not only on the nature of the ligand and the length of receptor stimulation, but also on the concurrent action of cytokines. TGF-β and IL-10 have been shown to decrease the expression of NKG2D and NKp30 [32,39,40]. Therefore, it can be reasoned that, in subjects infected with *T. cruzi* who show no signs of heart disease, NK-cell function may be balanced by the downregulation of activating receptors and IL-10 production. Herein, we have demonstrated that NK cells in *T. cruzi*-infected individuals without heart disease exhibit an increased presence of IL-10-producing NK cells.

Due to the prolonged nature of chronic *T. cruzi* infection, it is likely that the downregulation of activating receptors and IL-10 production is disrupted. This means that NK cells become dependent on direct contact with target cells for activation and subsequent potent cytotoxicity, a hypothesis that is consistent with our findings [41,42]. Other studies have shown a positive correlation between IL-10 and IFN-γ levels in the serum of subjects infected with *T. cruzi* without heart disease, whereas this correlation was not observed in patients with advanced cardiomyopathy. [43]. Overall, the NK cells of symptomatic patients are not exhausted, but rather unregulated.

We have demonstrated that, in patients with more advanced heart disease, T cells lose the ability to regulate IL-7R components [44,45], which is associated with a decrease in *T. cruzi*-specific IFN-γ-producing T cells [24,45]. This raises the question of whether, as with other chronic infections, NK cells are also involved in impairing parasite-specific T cells in chronic Chagas disease [13]. Infection with a virulent *T. cruzi* strain has been shown to alter NK cell-mediated regulation of the adaptive immune response induced by dendritic cells in an experimental infection in mice [46]. Studies performed on subjects with chronic *T. cruzi* infection support that NK cells not only have a regulatory function [47,48], but also increased expression of genes associated with cytotoxic and apoptotic responses of these cells was associated with heart disease [49,50]. A recent study supports that NK CD56^dim^ cells are highly found in the left ventricle of patients with idiopathic dilated cardiomyopathy associated to high expressions of granzyme B and other genes related with cytotoxicity [51]. However, other studies have shown that NK cells might limit cardiac inflammation and fibrosis [52]. In the present study, NK cells in subjects without heart disease appeared to positively regulate *T. cruzi*-specific T cell responses, as evidenced by the decrease in *T. cruzi*-specific IFN-γ- and IL-2-producing cells following CD56 depletion in this patient group.

Although CD56^+^ cell depletion was incomplete, we demonstrated that, following the depletion of CD4^+^ and CD8^+^ cells, samples enriched in CD56^+^ cells did not produce IFN-γ in response to *T. cruzi* antigens. Therefore, T cells were the primary source of IFN-γ-producing cells, as previously reported [24]. It is more likely that NK-antigen presenting cell cross-talk and cytokine-dependent mechanisms (e.g., IL-12/IL-18) mediate NK-cell activation, which in turn promotes parasite-specific T-cell responses [41,42]. Several mechanisms by which NK cells promote T-cell responses have been described, involving co-stimulatory molecules, cytokine secretion, and improved antigen presentation [41,42]. In addition to NK cells other immunoregulatory mechanisms may shape T-cell responses in individuals without heart disease, as high levels of Tregs and IL-10-producing CD4^+^ T cells have been observed in this patient group compared to those with advanced cardiomyopathy [53,54]. Since CD56-depletion did not discriminate between NK and NKT cells and despite NKT cells being a minor cell subset, we cannot rule out a positive effect of NKT cells on *T. cruzi*-specific T-cell responses [55]. However, direct functional validation through NK-T cell interaction assays will further confirm the effect of NK cells on parasite-specific T-cell function.

Treatment with benznidazole modulates NK-cell function, with declining *T. cruzi*-specific antibodies associated with monofunctional TNF-α-producing NK cells. This supports the idea that a more quiescent NK-cell function is induced upon *T. cruzi* clearance. These results are consistent with studies in adult subjects showing that the function of memory CD4^+^ T cells improves after benznidazole treatment [56,57], alongside a decline in inflammatory cytokines [44,57].

One limitation of the study is the small number of samples in the cardiac disease and treatment groups. The G2/G3 also has a higher proportion of male subjects than female subjects. Accordingly, cardiomyopathy in the chronic phase of *T. cruzi* infection is more frequently observed in males while females show slower progression [58]. Therefore, gender imbalance may be a potential confounding factor that could be addressed in future studies with larger sample sizes. The findings of this study may have therapeutic implications, as regulating inflammation could strike a balance between clearing infections and causing tissue damage. This may be particularly important for older patients, who may have additional health conditions such as atherosclerosis and coronary artery disease.

## Supporting information

S1 Table Antibodies used for surface and intracellular staining assays by flow cytometry. (DOCX)

S2 Table The frequency of NK-cell subsets in subjects with chronic Chagas disease. PBMC samples from *T. cruzi* infected subjects with no signs (G0, n = 15), with mild (G1, n = 13) or severe (G2-G3, n = 9) heart disease and uninfected controls (UN, n = 15) were stained with a panel of monoclonal antibodies specific for NK cells as described in Material and Methods and analyzed by flow cytometry. The frequency of CD56^bright^CD16^+/—^ and CD56^dim^CD16^+/—^ NK-cell subsets as a proportion of total NK cells are shown Differences among groups were assessed using a Kruskal‒Wallis test or ANOVA, with P values adjusted for multiple comparisons using Dunn’s or Holm‒Sidak posttest.
(DOCX)

S3 Table The phenotype and function of CD56^bright^ clusters identified by Phenograph and FlowSOM algorithm in subjects with chronic Chagas disease. Description of each cluster's function based on the expression (+) or non-expression (-) patterns of different markers.
(DOCX)

S4 Table The phenotype and function of CD56^dim^ clusters identified by Phenograph and FlowSOM algorithm in subjects with chronic Chagas disease. Description of each cluster's function based on the expression (+) or non-expression (-) patterns of different markers.
(DOCX)

S5 Table Percentages of individual CD56^bright^ and CD56^dim^ clusters identified by unsupervised high-dimensional analysis. Phenograph followed by FlowSOM clustering algorithms was applied on PBMC samples (G0, n = 11; G1, n = 7; G2-G3, n = 9; uninfected controls, UI, n = 15).Data are show as median percentages and range in each group of CD56^bright^ or CD56^dim^ clusters. Differences among groups were evaluated by ANOVA or the Kruskal–Wallis test followed by posttests.
(DOCX)

S6 Table Expression of the mean fluorescence intensity (MFI) for individual surface markers of the indicated cluster identified by unsupervised high-dimensional analysis. Phenograph followed by FlowSOM clustering algorithms was applied on PBMC samples (G0, n = 11; G1, n = 7; G2-G3, n = 9; uninfected controls, UI, n = 15). Data are show as median (range) expression of the mean fluorescence intensity (MFI) for individual surface markers. Differences among groups were evaluated by ANOVA or the Kruskal–Wallis test followed by posttests.
(DOCX)

S7 Table Manual gating strategy for the identification of NK cells with activating and cytotoxic phenotype. PBMC from *T. cruzi*-infected subjects (G0, n = 16; G1, n = 13; G2-G3, n = 9) and uninfected controls (UI, n = 15) were stained with a panel of monoclonal antibodies specific for NK cell surface markers and adquired by flow cytometry. Manual gating was used to select CD56^bright^CD16^—^and CD56^dim^CD16^+^ NK cells, which express NKp46/NKG2D but not CD57. Data are presented as median (range). Differences among groups were evaluated by ANOVA or the Kruskal–Wallis test followed by posttests.
(DOCX)

S8 Table Percentage of the total response contributed by CD56^+^ NK cells with given co-expression profile in subjects chronically infected with *T. cruzi.* PBMC of *T. cruzi*-infected subjects in the G0 (n = 16), G1 (n = 13), G2/G3 (n = 9) clinical groups, as well as from uninfected subjects (UI, n = 15), were stimulated with PMA/ionomycin for 4 h and analyzed by flow cytometry for intracellular expression of CD107a, granzyme B, IFN-γ, IL-10 and TNF-α. The data is show as median % (range) of the total response contributed by CD56^+^ NK cells with a given co-expression profile. Differences among groups were assessed using a Kruskal–Wallis test, with p values adjusted for multiple comparisons using Dunn's posttest.
(DOCX)

S1 Fig Gating strategy for NK cells in a representative donor. PBMC were stained for Zombie Aqua fixable viability stain and a combination of monoclonal antibodies anti-CD3 PerCP, anti-CD14 APC-Cy7, anti-CD16 BV785, CD19 APC-Cy7 and anti-CD56 BV605 and analyzed by flow cytometry. Lymphocytes were gated based on forward scatter area and side scatter area parameters, followed by forward scatter area vs. forward scatter height parameters and side scatter area vs. side scatter weight for doublet discrimination (single cells). Subsequent analyses were performed on viable (Zombie Aqua^—^) and CD14^—^CD19^—^CD3^—^CD56^+^ cells to identify CD56^bright^CD16^+/—^ and CD56^dim^CD16^+/—^ NK cells.
(DOCX)

S2 Fig Depletion of human CD56^+^ NK cells from PBMC derived from a *T. cruzi* infected donor. The PBMC were labelled with BD IMag anti-Human CD56 magnetic particles, separated using the BD IMag cell separation magnet and the negative (CD56^—^) fraction was collected. The process was repeated, after which the whole PBMC and the negative fractions of the first and second depletion steps were stained with anti-human CD56. The percentage of CD56^+^ cells is shown.
(DOCX)

S3 Fig Human CD4^+^ and CD8^+^ T cells were depleted from peripheral blood PBMC derived from one *T. cruzi*-infected donor. The PBMC were labelled with a mixture of BD IMag anti-human CD4 and CD8 magnetic particles, separated using a BD IMag cell separation magnet and the negative (CD4^—^ and CD8^—^) fractions were collected. The process was repeated, after which the whole PBMC and the negative fractions of the first and second depletion steps were stained with anti-human monoclonal antibodies specific for CD4, CD8 and CD3. The percentages of CD56^+^CD3^—^, CD56^+^CD3^+^, CD4^+^CD3^+^ and CD8^+^CD3^+^ cells are displayed. The numbers in brackets indicate the percentage depletion of the corresponding cell population.
(DOCX)

S4 Fig The frequency of NK-cell subsets in subjects with chronic Chagas disease. PBMC samples from *T. cruzi* infected subjects with no signs (G0, n = 15), with mild (G1, n = 13) or severe (G2-G3, n = 9) heart disease and uninfected controls (UN, n = 15) were stained with a panel of monoclonal antibodies specific for NK cells as described in Material and Methods and analyzed by flow cytometry. The frequency of CD56^bright^CD16^+/—^ (A) and CD56^dim^CD16^+/—^ (B) NK-cell subsets as a proportion of total NK cells are shown. Each symbol represents the value for a single subject. The bars indicate the median values and the interquartile range. Differences among groups were assessed using a Kruskal‒Wallis test or ANOVA, with P values adjusted for multiple comparisons using Dunn’s or Holm‒Sidak posttest. **** P < 0.0001, ** P < 0.01 and * P < 0.05.
(DOCX)

S5 Fig Representative manual gating strategy for the expression of NK activating receptors in subjects with chronic Chagas disease. PBMC from one *T. cruzi*-infected subject without heart disease (i.e., G0 clinical group A–D) or with severe cardiomyopathy (i.e., G3 clinical group E–H) were stained with a panel of monoclonal antibodies specific for the NK-cell lineage in combination with markers of NK cell activation, differentiation, immune inhibition and exhaustion, and acquired by flow cytometry, as described in the Materials and Methods section. A. CD56^bright^CD16^+/—^ and CD56^dim^CD16^+/—^ NK cells were selected as described in S1B Fig. NKp46^+^CD57^—^ cells were selected from CD56^bright^CD16^+^ cells. C. NKp46^+^CD57^—^ cells were selected from CD56^dim^CD16^+^ cells followed by expression of NKG2D (D).The percentages of selected cell populations are shown.
(DOCX)
