# Correction: Natural killer (NK) cells with downregulated activating receptors and IL-10 production promote *Trypanosoma cruzi* T-cell responses in subjects in the Chronic phase of *Trypanosoma cruzi* infection: An exploratory immunological analysis

**DOI:** 10.1371/journal.pntd.0014717

**Published:** 2026-09-14

**Authors:** María J. Elias, Gonzalo Cesar, María G. Alvarez, Ariel Podhorzer, María B. Caputo, Constanza Lopez Albizu, Máximo Carrega, Flavio A. Tomán Conte, María A. Natale, María C. Albareda, Bruno Lococo, Susana A. Laucella

Fig 5 was uploaded incorrectly. Please see the correct Fig 5 here.

An additional affiliation is missing for the second author. Gonzalo Cesar is also affiliated with #1: Instituto Nacional de Parasitología “Dr. Mario Fatala Chaben”, Buenos Aires, Argentina.

In the Results section, the subsection title “Contribution of NK cells to the T-cell response specific for T. cruzi,” was formatted incorrectly. The correct subsection title is: Contribution of NK cells to the T-cell response specific for *T. cruzi.*

**Fig 5:**
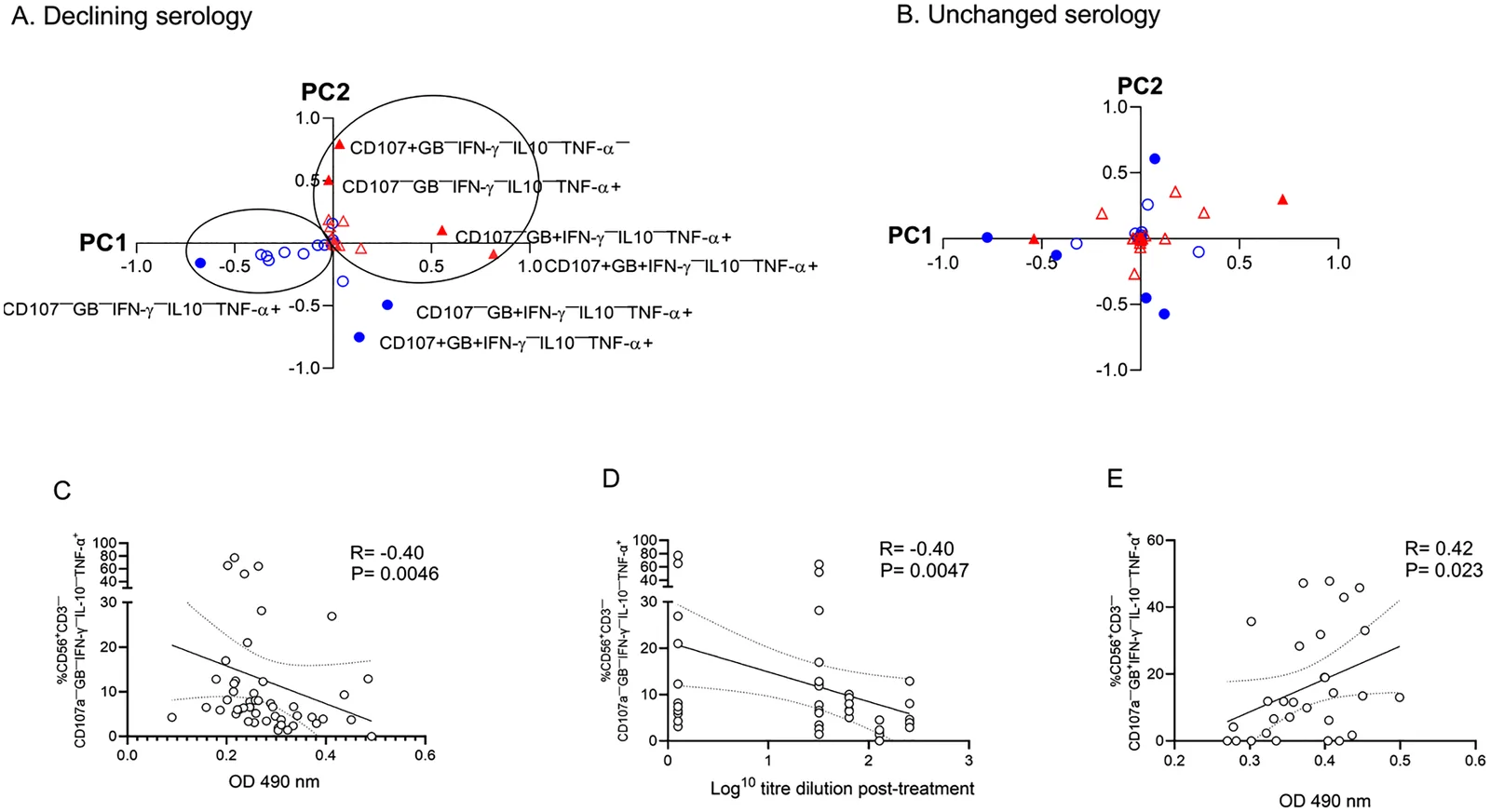
Principal component analysis of NK-cell function in *T. cruzi*-infected subjects following benznidazole treatment. PBMC were stimulated with PMA/ionomycin for 4 h and analyzed by flow cytometry for intracellular expression of CD107a, granzyme B, IFN-γ, IL-10 and TNF-α. Coexpression profiles with one to five functions using the Boolean gating function of FlowJo software. The contribution of each molecule-producing subset to the total NK-cell response was assessed in donors with positive responses in the intracellular staining assay as indicated in the Materials and Methods. Then, the average for each combination was calculated. The values obtained in unstimulated samples were subtracted. Following log transformation, the principal components were extracted with eigenvalues of 1.0. Factor loadings >0, 4 or <-0.4 were considered as influential mediators (filled symbols). NK cell functional profiles plotted based on the first 2 extracted principal components PC1 and PC2 prior to treatment (circle symbols) or following 48 months post-treatment with benznidazole (triangle symbols). *T. cruzi*-infected subjects with declining (A, n = 8) and or unchanged (B, n = 5) *T. cruzi*-specific antibodies following 48 months post-treatment. The Spearman test was applied to assess the correlation between the percentages of monofunctional and polyfunctional CD56^+^CD3-CD107-GB-IFN-gamma-IL-10-TNF-alpha^+—^ cells and *T. cruzi*-specific antibody levels measured by ELISA (C and E) and hemagglutination (D) in subjects with declining (C and D) or unchanged (E) serology for *T. cruzi* infection posttreatment.

## References

[1] Elias MJ, Cesar G, Alvarez MG, Podhorzer A, Caputo MB, Lopez Albizu C, et al. Natural killer (NK) cells with downregulated activating receptors and IL-10 production promote *Trypanosoma cruzi* T-cell responses in subjects in the Chronic phase of *Trypanosoma cruzi* infection: An exploratory immunological analysis. PLoS Negl Trop Dis. 2026;20(8):e0014622. doi: 10.1371/journal.pntd.0014622 42585265 PMC13489522

