# Supplementary material for: Natural killer (NK) cells with downregulated activating receptors and IL-10 production promote Trypanosoma cruzi T-cell responses in subjects in the Chronic phase of Trypanosoma cruzi infection: An exploratory immunological analysis
Source: PLoS Negl Trop Dis. 2026 Aug 12;20(8):e0014622. doi: 10.1371/journal.pntd.0014622 (PMC13489522; doi:10.1371/journal.pntd.0014622)
Supplement: Supplementary file 1 [file pntd.0014622.s001.docx]

S1 Table. Antibodies used for surface and intracellular staining assays by flow cytometry.

| Anti-human | Fluorohrome^a^ | Clone |
| --- | --- | --- |
|  |  |  |
| CD3 | PerCP | HIT3a |
| CD14 | APC-Cy7 | 63D3 |
| CD16 | BV785 | 3G8 |
| CD19 | APC-Cy7 | HIB19 |
| CD56 | BV605 | 5.1H11 |
| CD57 | PE | HNK-1 |
| CD107α | BV650 | H4A3 |
| CD127 | BV711 | A019D5 |
| CD158ß (KIR2DL2) | PE-Cy7 | DX27 |
| CD279 (PD1) | BV650 | EH12.2H7 |
| CD314 (NKG2D) | FITC | 1D11 |
| CD335 (NKp46) | APC | 9E2 |
| Granzyme B | FITC | QA16A02 |
| IFN-γ | PE-Cy7 | 4S.B3 |
| IL-10 | BV421 | JES3-9DY |
| TNF-α | APC | MAb11 |
| Zombie aqua | N/A | N/A |

^a^ APC, Allophycocyanin; APC-Cy7, Allophycocyanin/Cyanine7; BV421, Brillant Violet 421, BV510, Brillant Violet 510; BV605, Brillant Violet 650; BV650, Brillant Violet 650; BV711, Brillant Violet 711; BV785, Brillant Violet 785; FITC, Fluorescein isothiocyanate; PE, Phycoerythrin; PE-Cy7, Phycoerythrin-Cyanine7; PerCP, Peridinin Chlorophyll.
