# Supplementary material for: Natural killer (NK) cells with downregulated activating receptors and IL-10 production promote Trypanosoma cruzi T-cell responses in subjects in the Chronic phase of Trypanosoma cruzi infection: An exploratory immunological analysis
Source: PLoS Negl Trop Dis. 2026 Aug 12;20(8):e0014622. doi: 10.1371/journal.pntd.0014622 (PMC13489522; doi:10.1371/journal.pntd.0014622)
Supplement: Supplementary file 2 [file pntd.0014622.s002.docx]

S2 Table. The frequency of NK-cell subsets in subjects with chronic Chagas disease.

| NK cell population | Median % (range) | | | |  | | P value |
| --- | --- | --- | --- | --- | --- | --- | --- |
|  | G0^a^ (n = 15) | G1^b^(n = 13) | G2-3^c^(n = 9) | UI^d^ (n=15) | | |  |
| CD56^brigh^ | 4.3 (1.7-13.3) | 3.3 (0.4-7.5) | 4.7(1.5-15) | 9.2(4.9-15) | | *a,b,c vs d P < 0.05* | |
| CD56^dim^ | 95.7(86.8-98.3) | 96.7 (92.5-99.2) | 95.9 (85-98.4) | 90.8 (82-95) | | *a,b,c vs d P < 0.05* | |
