# Supplementary material for: Natural killer (NK) cells with downregulated activating receptors and IL-10 production promote Trypanosoma cruzi T-cell responses in subjects in the Chronic phase of Trypanosoma cruzi infection: An exploratory immunological analysis
Source: PLoS Negl Trop Dis. 2026 Aug 12;20(8):e0014622. doi: 10.1371/journal.pntd.0014622 (PMC13489522; doi:10.1371/journal.pntd.0014622)
Supplement: Supplementary file 4 [file pntd.0014622.s004.docx]

S4 Table. The phenotype and function of CD56^dim^ clusters identified by Phenograph

and FlowSOM algorithm in subjects with chronic Chagas disease.

| Cluster ID | Surface marker | | | | | | | Function |
| --- | --- | --- | --- | --- | --- | --- | --- | --- |
|  | NKp46 | PD1 | CD127 | CD16 | NKG2D | CD57 | KIRD2L |  |
| 1 | - | - | - | ++++ | + | ++ | - | Cytotoxicity, activation and differentiation |
| 2 | - | - | - | +++ | + | - | - | Activation and cytotoxicity |
| 4 | - | - | - | ++++ | + | + | + | Cytotoxicity, activation, differentiation and inhibition |
| 6 | - | - | - | +++ | + | - | + | Cytotoxicity, activation and inhibition |
| 11/14 |  | - | - | ++++ | + | - | - | Activation and cytotoxicity |
| 16 | - | - | - | ++ | + | - | - | Activation and cytotoxicity |
| 17 | - | - | - | - | + | - | - | Activation |
| 18 | + | - | - | +++ | + | - | - | Activation and cytotoxicity |
