# Supplementary material for: Natural killer (NK) cells with downregulated activating receptors and IL-10 production promote Trypanosoma cruzi T-cell responses in subjects in the Chronic phase of Trypanosoma cruzi infection: An exploratory immunological analysis
Source: PLoS Negl Trop Dis. 2026 Aug 12;20(8):e0014622. doi: 10.1371/journal.pntd.0014622 (PMC13489522; doi:10.1371/journal.pntd.0014622)
Supplement: Supplementary file 5 [file pntd.0014622.s005.docx]

S5 Table. Percentages of individual CD56^bright^ and CD56^dim^ clusters identified by unsupervised high-dimensional analysis.

| Cluster ID | | Median % (range) | | | | | P value |
| --- | --- | --- | --- | --- | --- | --- | --- |
|  | | G0^a^ (n = 11) | G1^b^ (n = 7) | | G2-3^c^ (n = 9) | UI^d^ (n=15) |  |
| **CD56 ^bright^** | |  |  | |  |  |  |
| Cluster 4 | 34.3 (14-49.7) | | 11.85 (6.3-15.7) | 11.3 (5.3-19.3) | | 18.7(8-25) | *b,c,d vs a P < 0.05* |
| Cluster 5 | | 1.7 (0-5) | 5.7 (1-10.3) | | 3.8 (1-10) | 3.7(0.3-10) | *a vs b P < 0.05* |
| **CD56^dim^** | |  |  | |  |  |  |
| Cluster 1 | | 5 (0.4-10.3) | 14 (4.5-29.3) | | 14 (3.6-29.3) | 1(0-20.4) | b,c vs d P< 0.05 |
| Cluster 4 | | 3.5 (0-19.6) | 5.4 (0.52-10.5) | | 4.4 (0.5-11.9) | 1(0.16-3.1) | *b vs d* *P < 0.05* |
| Cluster 11 | | 20.9 (3.9-56) | 21.6 (6.6-44.4) | | 32.6 (15.1-57) | 62.2 (31.4-70.2) | a,b,c vs d P < 0.05 |
| Cluster 18 | | 1 (0.08-3.1) | 1.5 (0.24-7.1) | | 3.8 (0.9-6.2) | 3.6 (1.9-19.2) | *a vs c,d P < 0.05* |
