# Supplementary material for: Natural killer (NK) cells with downregulated activating receptors and IL-10 production promote Trypanosoma cruzi T-cell responses in subjects in the Chronic phase of Trypanosoma cruzi infection: An exploratory immunological analysis
Source: PLoS Negl Trop Dis. 2026 Aug 12;20(8):e0014622. doi: 10.1371/journal.pntd.0014622 (PMC13489522; doi:10.1371/journal.pntd.0014622)
Supplement: Supplementary file 6 [file pntd.0014622.s006.docx]

S6 Table. Expression of the mean fluorescence intensity (MFI) for individual surface markers of the indicated cluster identified by unsupervised high-dimensional analysis.

| **Marker ID on CD56^bright^** | Median MIF (range) | | | | P value |
| --- | --- | --- | --- | --- | --- |
|  | G0^a^ (n = 12) | G1^b^ (n = 7) | G2-3^c^ (n = 9) | UI^d^ (n=15) |  |
| NKp46 of cluster 1 | 1064 (657-1661) | 1917 (1275-2314) | 1972 (1302-2681) | 1674 (1381-2275) | a vs b,c,d P*< 0.05* |
|  |  |  |  |  |  |
| NKp46 of cluster 4 | 622 (363-796) | 621 (467-858) | 998 (781-1132) | 962 (697-1204) | a,b vs d P*< 0.05;a,b vs c*  P*< 0.05* |
| **Marker ID on CD56^dim^** |  |  |  |  |  |
| CD16 of cluster 6 | 6129 (4041-12553) | 8562 (4976-16382) | 9420 (5989-18030) | 10041 (6777-14184) | a vs d P*< 0.05* |
|  |  |  |  |  |  |
| NKp46 of cluster 18 | 2025 (1835-2279) | 2372 (2156-2519) | 2391 (1957-2871) | 2270 (1991-2539) | a vs b,c,d P*< 0.05* |
|  |  |  |  |  |  |
