# Supplementary material for: Natural killer (NK) cells with downregulated activating receptors and IL-10 production promote Trypanosoma cruzi T-cell responses in subjects in the Chronic phase of Trypanosoma cruzi infection: An exploratory immunological analysis
Source: PLoS Negl Trop Dis. 2026 Aug 12;20(8):e0014622. doi: 10.1371/journal.pntd.0014622 (PMC13489522; doi:10.1371/journal.pntd.0014622)
Supplement: Supplementary file 7 [file pntd.0014622.s007.docx]

S7 Table . Manual gating strategy for the identification of NK cells with activating and cytotoxic phenotype

| NK-cell population | | Median % (range) | | |  | P value |
| --- | --- | --- | --- | --- | --- | --- |
|  | | G0^a^ (n = 16) | G1^b^ (n = 13) | G2-3^c^(n = 9) | UI^d^ (n=15) |  |
| CD56^bright^CD16^+^  NKp46^+^CD57^—^ | 48.45 (29-81.7) | | 37.00 (0-90.3) | 70.90 (43.9-91.3) | 81.30(36.6-92) | *a,b,c vs d P < 0.05* |
|  | |  |  |  |  |  |
| CD56^dim^CD16^+^NKp46^+^  NKG2D^+^CD57^—^ | | 36.15(15.4-65.7) | 49.40 (0-100) | 56.20 (34.7-94.1) | 70.60 (0-100) | *b vs e P < 0.05; b vs d P< 0.01* |
