# Supplementary material for: Natural killer (NK) cells with downregulated activating receptors and IL-10 production promote Trypanosoma cruzi T-cell responses in subjects in the Chronic phase of Trypanosoma cruzi infection: An exploratory immunological analysis
Source: PLoS Negl Trop Dis. 2026 Aug 12;20(8):e0014622. doi: 10.1371/journal.pntd.0014622 (PMC13489522; doi:10.1371/journal.pntd.0014622)
Supplement: Supplementary file 8 [file pntd.0014622.s008.docx]

S8 Table. Percentage of the total response contributed by CD56^+^ NK cells with given co expression profile in subjects chronically infected with *T. cruzi*

| NK-cell population | Median % (range) | | | | P value |
| --- | --- | --- | --- | --- | --- |
|  | G0^a^ | G1^b^ | G2-G3^c^ | UI^d^ |  |
| CD107a^+^Granzyme^+^IFN-γ^—^ IL10^—^ TNF-α^+^ | 0.61 (0-26.26) | 5 (0-48.9) | 6.7 (0-48.7) | 0.5 (0-12.51) | *a vs b P < 0.05: d vs b P < 0.05* |
|  |  |  |  |  |  |
| CD107a^—^ Granzyme^+^ IFN-γ^—^ IL10^+^TNF-α^—^ | 0 (0-90.7) | 0 (0-19) | 0 (0-46) | 0 (0-75.6) | b.c vs d *P < 0.05; a vs b,c P < 0.05* |
|  |  |  |  |  |  |
| CD107a^—^ Granzyme^+^ IFN-γ^—^ IL10^—^ TNF-α^+^ | 3.5 (0-82.7) | 12 (0-81) | 30.5 (4.5-51.7) | 0 (0-59) | a vs c, d *P < 0.05; d vs b,c P < 0.05* |
|  |  |  |  |  |  |
| CD107a^+^Granzyme^—^  IFN-γ^—^ IL10^—^ TNF-α^—^ | 2.8 (0-56) | 7.2 (0-31.2) | 4.3 (0-25) | 0.26 (0-85.4) | c vs d *P < 0.05* |
|  |  |  |  |  |  |
| CD107a^—^ Granzyme^—^  IFN-γ^—^ IL10^+^TNF-α^—^ | 0.66 (0-24.4) | 0.15 (0-5.6) | 0 (0-37.2) | 1.7 (0-54.5) | b vs d *P < 0.05* |
