# Supplementary material for: Natural killer (NK) cells with downregulated activating receptors and IL-10 production promote Trypanosoma cruzi T-cell responses in subjects in the Chronic phase of Trypanosoma cruzi infection: An exploratory immunological analysis
Source: PLoS Negl Trop Dis. 2026 Aug 12;20(8):e0014622. doi: 10.1371/journal.pntd.0014622 (PMC13489522; doi:10.1371/journal.pntd.0014622)
Supplement: Supplementary file 9 [file pntd.0014622.s009.docx]

Supplementary Figure 1. Gating strategy for NK cells in a representative donor. PBMC were stained for Zombie Aqua fixable viability stain and a combination of monoclonal antibodies anti-CD3 PerCP, anti-CD14 APC-Cy7, anti-CD16 BV785, CD19 APC-Cy7 and anti-CD56 BV605 and analyzed by flow cytometry. Lymphocytes were gated based on forward scatter area and side scatter area parameters, followed by forward scatter area vs. forward scatter height parameters and side scatter area vs. side scatter weight for doublet discrimination (single cells). Subsequent analyses were performed on viable (Zombie Aqua^—^) and CD14^—^CD19^—^CD3^—^CD56^+^ cells to identify CD56^bright^CD16^+/—^ and CD56^dim^CD16^+/—^ NK cells.
