# Supplementary material for: Natural killer (NK) cells with downregulated activating receptors and IL-10 production promote Trypanosoma cruzi T-cell responses in subjects in the Chronic phase of Trypanosoma cruzi infection: An exploratory immunological analysis
Source: PLoS Negl Trop Dis. 2026 Aug 12;20(8):e0014622. doi: 10.1371/journal.pntd.0014622 (PMC13489522; doi:10.1371/journal.pntd.0014622)
Supplement: Supplementary file 10 [file pntd.0014622.s010.docx]

S2 Fig : Depletion of human CD56^+^ NK cells from PBMC derived from a *T. cruzi* infected donor. The PBMC were labelled with BD IMag anti-Human CD56 magnetic particles, separated using the BD IMag cell separation magnet and the negative (CD56^—^) fraction was collected. The process was repeated, after which the whole PBMC and the negative fractions of the first and second depletion steps were stained with anti-human CD56. The percentage of CD56^+^ cells is shown.
