# Supplementary material for: Natural killer (NK) cells with downregulated activating receptors and IL-10 production promote Trypanosoma cruzi T-cell responses in subjects in the Chronic phase of Trypanosoma cruzi infection: An exploratory immunological analysis
Source: PLoS Negl Trop Dis. 2026 Aug 12;20(8):e0014622. doi: 10.1371/journal.pntd.0014622 (PMC13489522; doi:10.1371/journal.pntd.0014622)
Supplement: Supplementary file 11 [file pntd.0014622.s011.docx]

S3 Fig. Human CD4^+^ and CD8^+^ T cells were depleted from peripheral blood PBMC derived from one *T. cruzi*-infected donor. The PBMC were labelled with a mixture of BD IMag anti-human CD4 and CD8 magnetic particles, separated using a BD IMag cell separation magnet and the negative (CD4^—^ and CD8^—^) fractions were collected. The process was repeated, after which the whole PBMC and the negative fractions of the first and second depletion steps were stained with anti-human monoclonal antibodies specific for CD4, CD8 and CD3. The percentages of CD56^+^CD3^—^, CD56^+^CD3^+^, CD4^+^CD3^+^ and CD8^+^CD3^+^ cells are displayed. The numbers in brackets indicate the percentage depletion of the corresponding cell population.
