# Supplementary material for: Natural killer (NK) cells with downregulated activating receptors and IL-10 production promote Trypanosoma cruzi T-cell responses in subjects in the Chronic phase of Trypanosoma cruzi infection: An exploratory immunological analysis
Source: PLoS Negl Trop Dis. 2026 Aug 12;20(8):e0014622. doi: 10.1371/journal.pntd.0014622 (PMC13489522; doi:10.1371/journal.pntd.0014622)
Supplement: Supplementary file 12 [file pntd.0014622.s012.docx]

S4 Fig. The frequency of NK-cell subsets in subjects with chronic Chagas disease. PBMC samples from *T. cruzi* infected subjects with no signs (G0, n = 15), with mild (G1, n = 13) or severe (G2-G3, n = 9) heart disease and uninfected controls (UN, n = 15) were stained with a panel of monoclonal antibodies specific for NK cells as described in Material and Methods and analyzed by flow cytometry. The frequency of CD56^bright^CD16^+/—^ (A) and CD56^dim^CD16^+/—^ (B) NK-cell subsets as a proportion of total NK cells are shown. Each symbol represents the value for a single subject. The bars indicate the median values and the interquartile range. Differences among groups were assessed using a Kruskal‒Wallis test or ANOVA, with P values adjusted for multiple comparisons using Dunn’s or Holm‒Sidak posttest. **** P < 0.0001, ** P < 0.01 and * P < 0.05.
