# Supplementary material for: Natural killer (NK) cells with downregulated activating receptors and IL-10 production promote Trypanosoma cruzi T-cell responses in subjects in the Chronic phase of Trypanosoma cruzi infection: An exploratory immunological analysis
Source: PLoS Negl Trop Dis. 2026 Aug 12;20(8):e0014622. doi: 10.1371/journal.pntd.0014622 (PMC13489522; doi:10.1371/journal.pntd.0014622)
Supplement: Supplementary file 13 [file pntd.0014622.s013.docx]

S5 Fig. Representative manual gating strategy for the expression of NK activating receptors in subjects with chronic Chagas disease. PBMC from one *T. cruzi*-infected subject without heart disease (i.e. G0 clinical group A–D) or with severe cardiomyopathy (i.e. G3 clinical group E–H) were stained with a panel of monoclonal antibodies specific for the NK-cell lineage in combination with markers of NK cell activation, differentiation, immune inhibition and exhaustion, and acquired by flow cytometry, as described in the Materials and Methods section. A. CD56^bright^CD16^+/—^ and CD56^dim^CD16^+/—^ NK cells were selected as described in Supplementary Figure 1. B. NKp46^+^CD57^—^ cells were selected from CD56^bright^CD16^+^ cells. C. NKp46^+^CD57^—^ cells were selected from CD56^dim^CD16^+^ cells followed by expression of NKG2D (D).The percentages of selected cell populations are shown.
